# Genomic Characterization of *Campylobacter jejuni* Adapted to the Guinea Pig (*Cavia porcellus*) Host

**DOI:** 10.3389/fcimb.2021.607747

**Published:** 2021-03-18

**Authors:** Craig T. Parker, Kerry K. Cooper, Francesca Schiaffino, William G. Miller, Steven Huynh, Hannah K. Gray, Maribel Paredes Olortegui, Paul Garcia Bardales, Dixner Rengifo Trigoso, Pablo Penataro-Yori, Margaret N. Kosek

**Affiliations:** ^1^ Produce Safety and Microbiology Research Unit, Agricultural Research Service, US Department of Agriculture, Albany, CA, United States; ^2^ School of Animal and Comparative Biomedical Sciences, University of Arizona, Tucson, AZ, United States; ^3^ Faculty of Veterinary Medicine, Universidad Peruana Cayetano Heredia, Lima, Peru; ^4^ The Division of Infectious Diseases and International Health and Public Health Sciences, University of Virginia, Charlottesville, VA, United States; ^5^ Whiting School of Engineering, Johns Hopkins University, Baltimore, MD, United States; ^6^ Biomedical Research, Asociación Benéfica PRISMA, Iquitos, Peru

**Keywords:** gastroenteritis, campylobacteriosis, *Campylobacter jejuni*, selenocysteine, source attribution

## Abstract

*Campylobacter jejuni* is the leading bacterial cause of gastroenteritis worldwide with excessive incidence in low-and middle-income countries (LMIC). During a survey for *C. jejuni* from putative animal hosts in a town in the Peruvian Amazon, we were able to isolate and whole genome sequence two *C. jejuni* strains from domesticated guinea pigs (*Cavia porcellus*). The *C. jejuni* isolated from guinea pigs had a novel multilocus sequence type that shared some alleles with other *C. jejuni* collected from guinea pigs. Average nucleotide identity and phylogenetic analysis with a collection of *C. jejuni* subsp*. jejuni* and *C. jejuni* subsp*. doylei* suggest that the guinea pig isolates are distinct. Genomic comparisons demonstrated gene gain and loss that could be associated with guinea pig host specialization related to guinea pig diet, anatomy, and physiology including the deletion of genes involved with selenium metabolism, including genes encoding the selenocysteine insertion machinery and selenocysteine-containing proteins.

## Introduction

The Gram-negative zoonotic bacteria *Campylobacter jejuni* is a principal cause of bacterial foodborne illness worldwide. *C. jejuni* is separated into two different sub-species, *C. jejuni* subsp. *jejuni* (*Cjj*) and *C. jejuni* subsp. *doylei* (*Cjd*). Of these *Cjj* is the most widely distributed subspecies commonly isolated from poultry and ruminants (Nauta et al., 2009; Kaakoush et al., 2015; Skarp et al., 2016; Mourkas et al., 2020). Contrarily, *Cjd* is infrequently isolated, and then, only directly from human bacteremia and gastroenteritis cases (Kasper and Dickgiesser, 1985; Steele et al., 1985). These two sub-species are phenotypically distinguishable by the inability of *Cjd* to reduce nitrate and growth instability at 42°C, and they are genetically distinct by several consistently conserved genomic features (Lastovica and Skirrow, 2000; Miller et al., 2007; Parker et al., 2007).

In high income countries, such as the United States, the most common source of *C. jejuni* infection are undercooked poultry products, but other sources include raw milk, contaminated water, and most recently, juvenile canines (Friedman et al., 2000; Centers for Disease C, Prevention, 2013; Pitkanen, 2013; Eurosurveillance Editorial Team, 2015; Kaakoush et al., 2015; Burakoff et al., 2018; Montgomery et al., 2018). In low- and middle-income countries (LMICs), such as Peru, where this pathogen remains one of the leading causes of bacterial gastroenteritis in children under the age of five, the epidemiology of *Campylobacter* remains understudied (Platts-Mills and Kosek, 2014; Platts-Mills et al., 2015). Although recent genomic studies have begun to unfold the relationship between poultry sources and human infections, this analysis is restricted by the number of *Campylobacter* genomes available for comparison (Pascoe et al., 2020). As a result, the exploration of additional *Campylobacter* hosts in LMICs is a pre-requisite for future disease control interventions.

Whole genome sequencing of *C. jejuni* has revealed evidence for lineages isolated from multiple species (generalists) and for lineages isolated from predominantly single host species (specialists) (Sheppard et al., 2011; Sheppard et al., 2014; Mourkas et al., 2020). For example, *C. jejuni* isolated from cattle predominately contained genes encoding vitamin B5 biosynthesis, while the genes were frequently absent from strains isolated from poultry (Sheppard et al., 2013). Aside from poultry and ruminants, *C. jejuni* has been detected and isolated from numerous different birds (Cody et al., 2015; Atterby et al., 2018; Lawton et al., 2018) and a variety of mammalian species, including but not limited to raccoons, rodents and lagomorphs (Graham et al., 2016; Mutschall et al., 2020). Among these host, wild birds are also often colonized by host specialist *C. jejuni* (Cody et al., 2015; Lawton et al., 2018; Atterby et al., 2018). Studies in Ecuador have found *C. jejuni* in domestic guinea pigs (*Cavia porcellus*) (Lowenstein et al., 2016; Toledo et al., 2017), and multilocus sequence typing (MLST) analysis of these isolates showed the presence of many unique sequence types (Graham et al., 2016). Among rural communities across Ecuador and Peru, guinea pigs are a substantial source of animal protein and are almost ubiquitously bred within communities located at high altitudes, however they are easily raised and commercialized throughout these countries.

In this study, we exploited whole genome sequencing (WGS) and comparative genomic analysis of *C. jejuni* isolates collected from guinea pigs, chickens and dogs in Santo Tomas, a town in the Peruvian Amazon, in order to ascertain possible sources of human infection. The isolates from the dogs and chickens were similar to *Cjj* isolated from human stool in this area of Peru. However, the analysis of genomes of *C. jejuni* strains isolated from guinea pigs provided evidence of considerable novel alterations including to gene gain and loss that presumably have allowed adaption to the guinea pig host.

## Materials and Methods

### Sampling and Culture

Guinea pig fecal samples were obtained from a guinea pig breeder located in Santo Tomas, Iquitos, Peru in the Peruvian Amazon. This is a peri-urban community of approximately 1,500 households made-up of ~5,000 individuals. Guinea pigs are a common source of animal protein in other areas of country, such as the highlands of Peru, yet not the Peruvian Amazon. Household rearing of guinea pigs in these communities are unusual, and this specific collection site was part of a multi-species farm individually rearing guinea pigs, goats and pigs for commercial activities. Approximately 150 guinea pigs were housed in a single pen without any other animal species.

Fecal pellets were taken and placed in Cary Blair transport medium and processed within 24 hours. Stools were inoculated on *Campylobacter* Blood Free Selective agar base (Oxoid, Lenexa, KS, USA) without any supplementation. Plates were incubated for 48 to 72 hours at 37°C at 5% O_2_, 10% CO_2_, 85% N_2_. Colonies demonstrating typical *Campylobacter* morphology were assessed using oxidase and catalase tests, as well as Gram staining. DNA was extracted from all bacterial cultures using PureLink Genomic DNA Mini Kit (Invitrogen, Carlsbad, CA, USA) as specified by manufacturer’s instructions. A duplex qPCR targeting a 16S rRNA and the *Campylobacter* adhesion to fibronectin (*cadF)* genes was performed to confirm all bacterial cultures as *Campylobacter* spp. or *C. jejuni*/*C. coli.*

### Bacterial Isolate Genome Sequencing

DNA was extracted from *C. jejuni* isolates (**Table 1**) and sequenced using an Illumina MiSeq platform. Sequencing libraries were prepared with the Nextera XT kit according to manufacturer’s instructions (Illumina, San Diego, CA), and batches of 24 isolate gDNA were barcoded and sequenced in multiplex to achieve 80-120x coverage. The pooled libraries were loaded into a MiSeq system and sequenced using a MiSeq reagent kit (v2, 500 cycle; Illumina). The sequence reads were trimmed and assembled using the SPAdes assembler (ver. 3.13.0) (Bankevich et al., 2012) within Geneious Prime 2020.2.1. The average number of contigs was 64 (range: 43–101) for an average total assembled sequence size of 1.67 Mbp (range: 1.61–1.74). The average N50 contig length (L50) was 14,577 bp (range: 3,794-55,912 bp) and the average GC content was 30.8% (range: 30.5-31.6). Short read data and assembled WGS are available on the NCBI SRA and NCBI WGS and are associated with BioProjects PRJNA658163, PRJNA658164, PRJNA658165, PRJNA658166, PRJNA658168, PRJNA658171, PRJNA658172, and PRJNA658173.

**Table 1:** *C. jejuni* strains collected in this study.

Allele^a^											
Strain	Genome size	aspA	glnA	gltA	glyA	pgm	tkt	uncA	ST	CC	Genome^b^
Guineapig012	1.679	544	**538**	638	**601**	**730**	816	464	10316		JACRSF000000000
Guineapig013	1.681	544	**538**	638	**601**	**730**	816	464	10316		JACRSG000000000
Dog014	1.737	7	84	5	10	11	3	6	1036	ST-353	JACRSH000000000
Dog015	1.734	7	84	5	10	11	3	6	1036	ST-353	JACRSI000000000
Dog017	1.637	2	4	5	25	1057	203	5	10311		JACRSJ000000000
Chick018	1.618	2	17	5	10	11	3	6	8741	ST-353	JACRSK000000000
Chick019	1.620	2	17	5	10	11	3	6	8741	ST-353	JACRSL000000000
Chick020	1.620	2	17	5	10	11	3	6	8741	ST-353	JACRSM000000000

a Allele numbers in bold were observed in guinea pig isolates from Ecuador column Genome^b^.

### Molecular Typing and Comparison

The isolate genomes were submitted to the pubMLST database (https://pubmlst.org/campylobacter/) for curation and analysis. MLST sequence types (STs) as described previously were assigned (Dingle et al., 2001; Jolley et al., 2018). The assigned *C. jejuni* STs were used to search the complete *C. jejuni* database (98,275 profiles present on August 1, 2020). Among the isolates in the database, isolates from a dataset used in a previous study in Ecuador were compared to the MLST data set that was determined here and initial relationships of MLST data were visualized as a minimum spanning tree using the GrapeTree plugin of pubMLST (Zhou et al., 2018) (data not shown).

### Phylogenetic Analysis

A selection of MLST alleles from 82 *Campylobacter* (**Table 1**, **Supplementary Table 1**), including the eight isolates sequenced in this study, 44 isolated from animals in Ecuador (Graham et al., 2016), 18 additional Peruvian clinical isolates (Pascoe et al., 2020), eight from *Cjj* isolates, three from *Cjd* isolates, and one from *C. coli* were examined to determine the phylogenetic relationship. The MLST allele sequences were concatenated, and then alignments of the concatenated sequences were performed using the MAFFT (Katoh and Standley, 2013) plugin of Geneious Prime 2020.2.1. The neighbor-joining dendrogram and phylogenetic analyses were performed using MEGA version 7 (Kumar et al., 2016). Briefly, the dendrogram was constructed using the neighbor-joining method and Poisson correction. Bootstraps were conducted with 500 replicates. Similarly, for isolates with WGS data, the sequences for 62 core genes (see **Figure 2**) were concatenated, and then alignments of the concatenated sequences were performed using the MAFFT. Thirty-eight *Campylobacter* genomes, including the eight isolates from this study, 18 additional Peruvian clinical isolates (Pascoe et al., 2020), eight complete genome *Cjj* strains, three from *Cjd* strains, and one from *C. coli* were examined to determine the phylogenetic relationship in more detail. For each genome, the sequences for 62 core genes (see **Figure 2**) were concatenated, and then alignments of the concatenated sequences were performed using the MAFFT (Katoh and Standley, 2013) plugin of Geneious Prime 2020.2.1. The neighbor-joining dendrogram and phylogenetic analyses were performed using MEGA version 7 (Kumar et al., 2016). Briefly, the dendrogram was constructed using the neighbor-joining method and Poisson correction. Bootstraps were conducted with 500 replicates.

### Pangenome Analysis

The thirty-eight *Campylobacter* strains selected for phylogenetic analysis were also utilized for the initial pangenome analysis to generate and visualize the Peruvian pangenome of *C. jejuni* compared to other well characterized *Campylobacter* strains using Anvi’o software (v6.2) (Eren et al., 2015) with the pangenomics workflow (Delmont and Eren, 2018). The species, subspecies and source for each of the strains was imported as additional layers in the database, and the average nucleotide identity (ANI) was generated using fastANI software (v1.31) (Jain et al., 2018). To visualize the different aspects of the pangenome, the gene clusters were binned using the search filters in the Anvi’o interactive interface using the following search conditions: (1) core genes – gene clusters present in minimum of 36 genomes (95%); (2) accessory genes – gene clusters present in minimum of 2 genomes and maximum of 35 genomes; (3) singleton genes – gene clusters present in a maximum of 1 genome using Anvi’o analysis blastp with a minimum bit score of 0.5 for gene clustering

### Core Genome Alignment Analysis

The thirty-eight *Campylobacter* strains described previously were also used to determine the core genome alignment using the Harvest software suite (Treangen et al., 2014). The core genome alignment was determined using Parsnp software (v1.1) with the -c flag (ignore MUMi) and the *C. jejuni* str. Guineapig012 as the reference genome. The Parsnp output was visualized with Gingr software (v1.2), and the newick file visualized with MEGA X software (Kumar et al., 2018) for final figure preparation. Additionally, the newick file was visualized with Anvi’o software with the following strain information added as an additional layer: (1) species; (2) subspecies; (3) source; (4) sequence type (ST); (5) clonal complex; (6) cgMLST; (7) percentage of CJIE1 present in genome; (8) percentage of CJIE2 present in genome; (9) percentage of CJIE3 present in genome; (10) percentage of CJIE4 present in genome. The sequence type (ST), clonal complex, and cgMLST for each of the strains was determined by screening each of the genomes against the pubMLST database (https://pubmlst.org/campylobacter/). The percentage of *Campylobacter jejuni* integrated elements (CJIE) 1-4 (CJIE1, CJIE2, CJIE3, and CJIE4) present in each of the genomes was determined by extracting the DNA sequence of each of the four CJIEs from *Campylobacter jejuni* subsp. *jejuni* str. RM1221 and conducting a BLASTn search against each of the genomes using Geneious Prime (v2020.1.2). Percentage represents total percentage of the particular CJIE present in the genome of that strain and is represented in the figure by intensity.

### Core and Accessory Genome Analysis

The initial pangenome analysis results generated by Anvi’o were confirmed using Roary and Scoary software. The core and accessory genome of 37 C*. jejuni* strains (the *C. coli* str. RM5611 was excluded from this analysis) were determined at 90% identity using Roary software (v3.12.0) (Page et al., 2015) with the following flags: -e (create a multiFASTA alignment of core genes using PRANK); -n (fast core gene alignment with MAFFT); -v (verbose output to STDOUT); -i 90 (minimum percentage identity for blastp; 90%). The Roary analysis was repeated at the 95% and 85% identity cutoffs to check for any major variations in the core and accessory genomes at the different percentages. The number of core, soft-core, shell and cloud genes as well as the overall core and accessory genome determined by the Roary analysis were visualized using the roary_plots.py script. Genes statistically unique to the two guinea pig associated *C. jejuni* genomes and those genes statistically significantly missing in the two genomes compared to the other 35 C*. jejuni* genomes were identified using the Scoary program (v1.6.16) (Brynildsrud et al., 2016) with the following command: scoary -g 90_gene_presence_absence. csv -t Guinea_pig_traits.csv -o Scoary/90_percent.

## Results

### *C. jejuni* Isolates From Guinea Pig Cluster With Others From the Same Host

**Figure 1:**
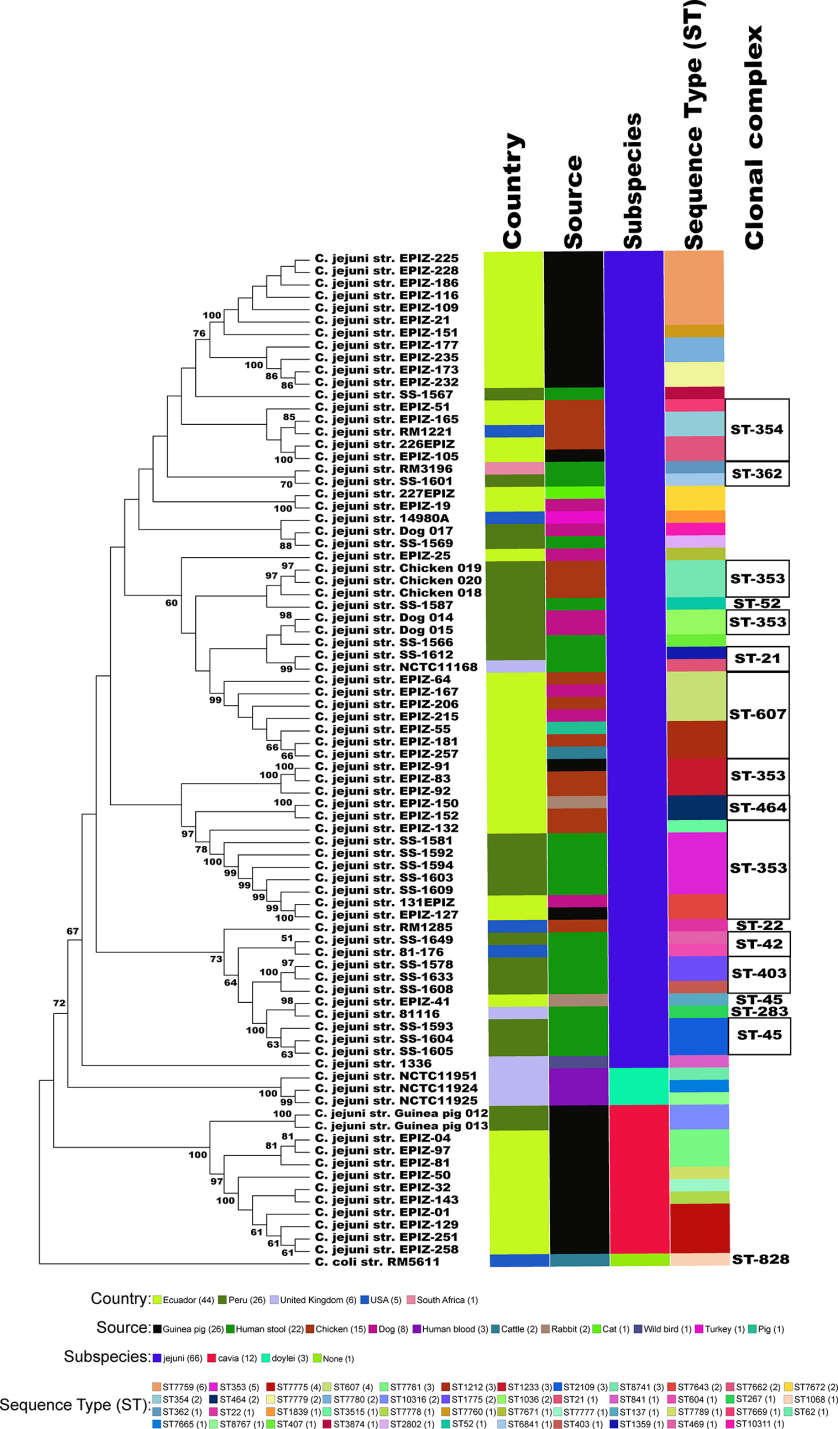
Minimum spanning tree of the MLST results. The concatenated sequences of the 7 MLST alleles (*aspA, glnA, gltA, glyA, pgm, tkt*, and *uncA*) from 82 Campylobacter strains were aligned with MAFFT and a dendrogam was created using neighbor-joining algorithm and the Kimura 2-parameter distance correction model. The concatenated profile sequence for the *C. coli* strain RM5611 (CP007179) was included for comparison. The topology only is shown in the figure. Metadata for isolates including country, isolate source, subspecies and sequence type are color-coded and noted in the key associated with the figure. Clonal complex is also noted in the figure.

We collected and sequenced *C. jejuni* isolates from domestic guinea pigs (n=2), domestic chickens (n=3) and domestic dogs (n=3) within Santo Tomas, Peru. The *C. jejuni* isolates were assigned four sequence types (ST) and 5 isolates shared a clonal complex (CC-353) (**Table 1**). The STs of the isolates from the dogs (ST-1036 and ST-10311) and chickens (ST-8741) were identified among several *C. jejuni* in the pubMLST database. Both guinea pig isolates possessed the same novel ST-10316, but some of its rare alleles (glnA538, glyA601 and pgm730) were present in other STs found in a group of guinea pigs isolates from Ecuador (**Supplementary Table 1**). We examined the relationship between these strains, along with a collection of Peruvian clinical *Cjj* strains (Pascoe et al., 2020), and well-studied *Cjj* strains with complete genomes, three *Cjd* strains, and a *C. coli* strain (**Supplementary Table 1**). As the Ecuadorian strains only had data for the 7 MLST gene alleles, the analysis only used the MLST allele sequences from all strains to create a neighbor joining dendrogram (**Figure 1**). The results clearly indicated that there were two large clusters of guinea pig *C. jejuni* isolates. The first cluster was within the *Cjj* clade, and the other cluster that contains the isolates from Peru formed a clade distinct from the other *C. jejuni* subspecies. Moreover, this clade was further away from the *Cjj* clade than the *Cjd* clade (**Figure 1**).

### Phylogenetic and ANI Analysis Support a New Subspecies for the Guinea Pig Isolates

**Figure 2:**
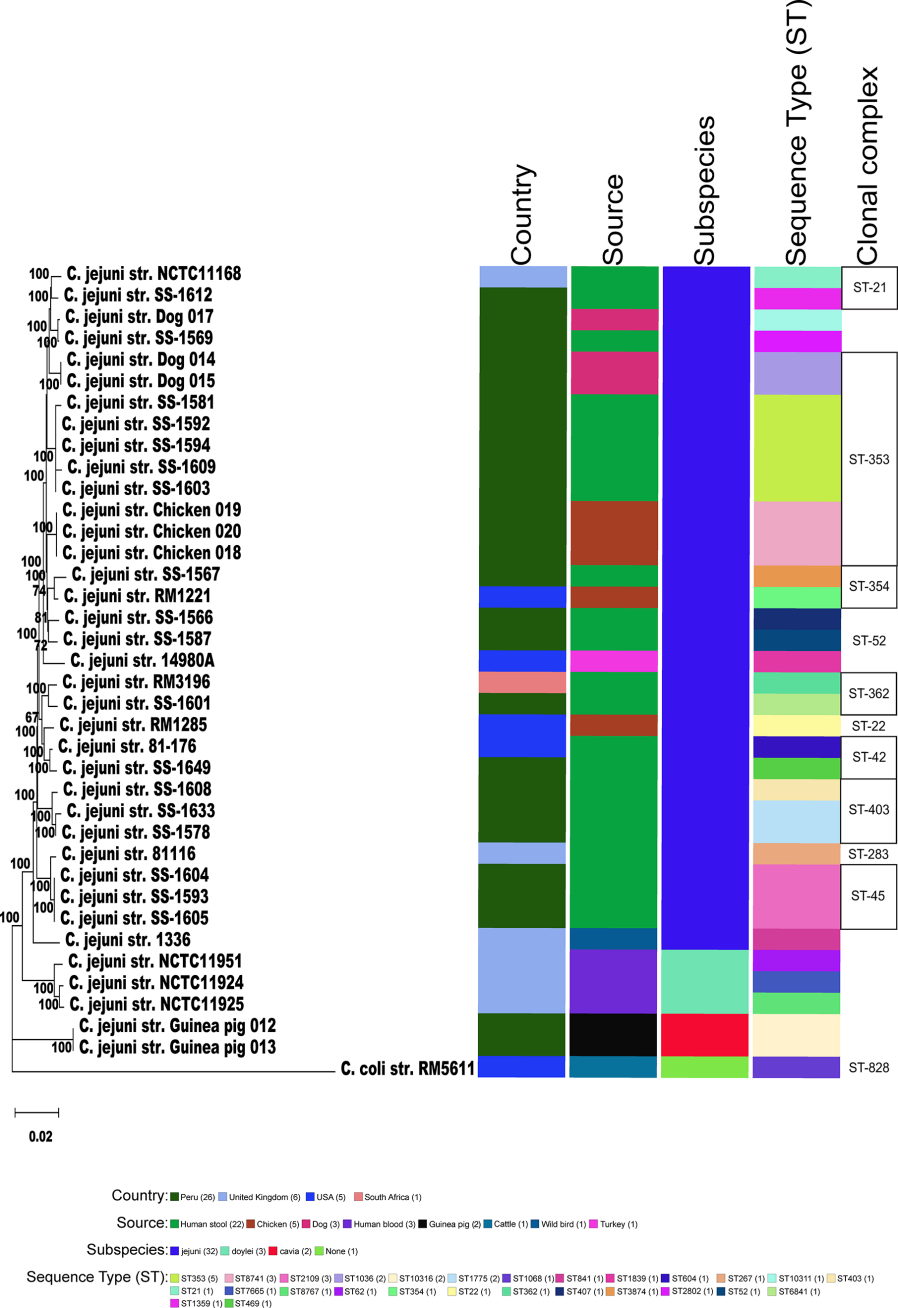
Phylogeny of Campylobacters. A) Sixty-two concatenated core genes (concatenated in the order gyrB, pyrG, aspA, atpA, infB, nrdB, lpxB, fabH, glmM, glyA, pgm, nusA, mqnC, clpB, tatC, kdsC, uvrB, glnA, dnaE, dnaK, msbA, dapA, fliP, trmA, folD, aroA, cheR, purH, argF, livM, cmeD, folC, pssA, waaC, dnaX, cfa, ftsY, groEL, pdxA, pnp, hydA, spoT, rodA, mobA, ppk, fumC, katA, fabI, kpsD, flgI, flgK, cadF, addB, putA, acs, nuoB, rplQ, tkt, recA, murB, gltA, secY) within the genomes of 38 genomic sequences were aligned with MAFFT. The dendrogram was constructed using the neighbor-joining algorithm and the Kimura 2-parameter distance correction model. The topology only is shown in the figure. Bootstrap values of ≥50%, generated from 500 replicates, are shown at the nodes. The concatenated profile sequence for the *C. coli* strain RM5611 (CP007179) was included for comparison. Metadata for isolates including country, isolate source, subspecies and sequence type are color-coded and noted in the key within the figure. Clonal complex is also noted in the figure.

**Figure 3:**
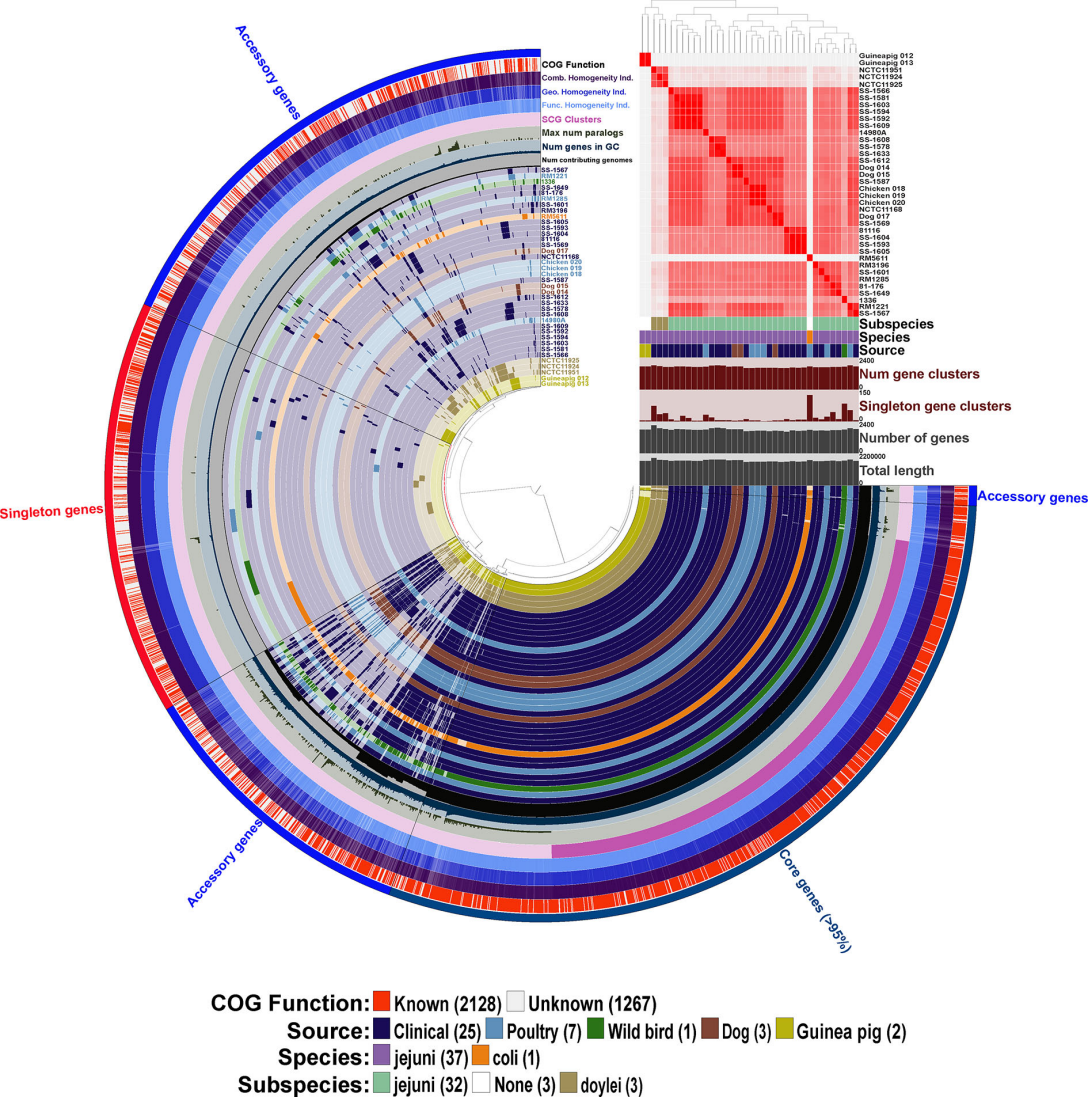
Initial pangenome analysis of *Campylobacter* strains isolated from various Peruvian sources and other previously characterized strains using Anvi’o software. Sample information includes the average nucleotide identity (ANI) for the 38 *Campylobacter* strains using percent identity for the intensity of each square with a >95% cutoff. Subspecies, species and source of isolation for each of the *Campylobacter* strains is included with the color key presented in the legend at the bottom of the figure, additionally the 38 strain layers or rings are colored corresponding to the source of isolation with the exception of the *C. coli* str. RM5611 (CP007179) (colored based on species) and the three *C. jejuni* subsp. *doylei* strains (colored based on subspecies). Sample information also includes for each individual strain the number of gene clusters, number of singleton gene clusters, total number of genes, and total length. The 38 inner rings represent the 38 *Campylobacter* strains and the presence/absence of a particular gene cluster with the dark colored bar representing the gene cluster is present versus the light-colored bar representing the gene cluster is absent from the strain. Dendrogram at the top of the figure organizes the 38 strain rings based on the frequency of 3,395 gene clusters determined in the pangenome analysis, whereas the inner dendrogram represents the relationship of the strains based on the presence/absence of the gene clusters. Each of the 3,395 gene clusters is binned into one of three following categories: (1) core genes (1,354 gene clusters; present in >95% of genomes); (2) accessory genes (1,344 gene clusters; present in 2 < genomes > 35); (3) singleton genes (697 gene cluster; present in only a single genome). The layer or ring immediately after the 38 *Campylobacter* strain layers represents the number of genomes contributing to each of the gene cluster bars in the 38 strain layers (ranging from 1 to 38 genomes). The next layer up signifies the number of genes in each of the gene clusters that the bars in the 38 strain layers represents, and the next two layers above is the maximum number of paralogs for each of the gene clusters and single copy gene clusters, respectively. The next three layers represent the homogeneity of the gene clusters including the functional homogeneity, geometric homogeneity and the combined homogeneity, respectively. The final outer layer displays if the cluster of orthologous groups (COG) function for the gene cluster is known or not, red representing a known function and white an unknown function. The figure and all described analysis was conducted using Anvi’o software (v6.2).

To better understand the relationship of the guinea pig isolates with other *C. jejuni*, phylogenetic analysis of those strains with WGS was performed using a set of 62 C*. jejuni/C. coli* core genes that were fairly evenly spaced around the chromosome. As previously mentioned, the genomic data for *C. jejuni* isolates from Ecuador only included the MLST alleles (Graham et al., 2016), and so, these samples were removed from further analysis. Again, the two guinea pig isolates from Peru formed a clade distinct from the other *C. jejuni* subspecies (**Figure 2**). The *C. jejuni* isolates collected from dogs and chickens, and all of the *C. jejuni* Peruvian clinical isolates examined were within the large *Cjj* clade. Average nucleotide identity (ANI) analysis of these genome sequences also provides evidence that the isolates from guinea pig were distinct with ANI values of ∼94-95% with both *Cjj* and *Cjd* genomes. Additionally, the ANI values between the two historic subspecies, *Cjj* and *Cjd*, is ∼95-96% (**Figure 3**).

### Genomic Features and Distinctions of the *C. jejuni* Isolated From Guinea Pig

The calculated genome sizes for the two isolates from guinea pig was ~ 1.68 Mb. This genome size is slightly larger than the mean genome size of 1.66 Mb (ranging from 1.61-1.79 Mb) for *Cjj* used in this study. In contrast, the genomes from guinea pig isolates were smaller than the three *Cjd* genomes that had a mean size of 1.79 Mb (ranging from 1.73-1.89 Mb).

We determined the pangenome using Anvi’o software for the 38 *Campylobacter* genomes and found a total of 3,395 gene clusters, where a cluster is a coding gene or a group of paralogous genes. The pangenome consisted of 1,354 clusters as part of the core genome (≥ 95% of genomes), 1,344 gene clusters in the accessory genome, and 697 gene clusters unique to a single genome (singleton). The composition of the gene clusters in the core genome were composed predominately of single copy genes compared to the vast majority of the gene clusters in the accessory genome containing paralogs. As a different species of *Campylobacter*, *C. coli* contained the largest amount of singleton gene clusters followed by *C. jejuni* subsp. *jejuni* str. 1336 (CM000854) that was isolated from a wild bird. Neither of the guinea pig isolates contained a large amount of singleton gene clusters particularly compared to *C. jejuni* subsp *doylei* strains and the *C. coli* strains, which is due to their similarity to each other. In fact, the two guinea pig isolates share a large unique portion of their genomes that are not found in the other 36 genomes in the analysis. Further examination of these guinea pig unique gene clusters shows that there are very few paralogs in the clusters, but there is a high level of geometric and functional homogeneity among the genes in the clusters. Although very few of the genes in these guinea pig unique gene clusters have an actual assigned COG function (**Figure 3**).

**Figure 4:**
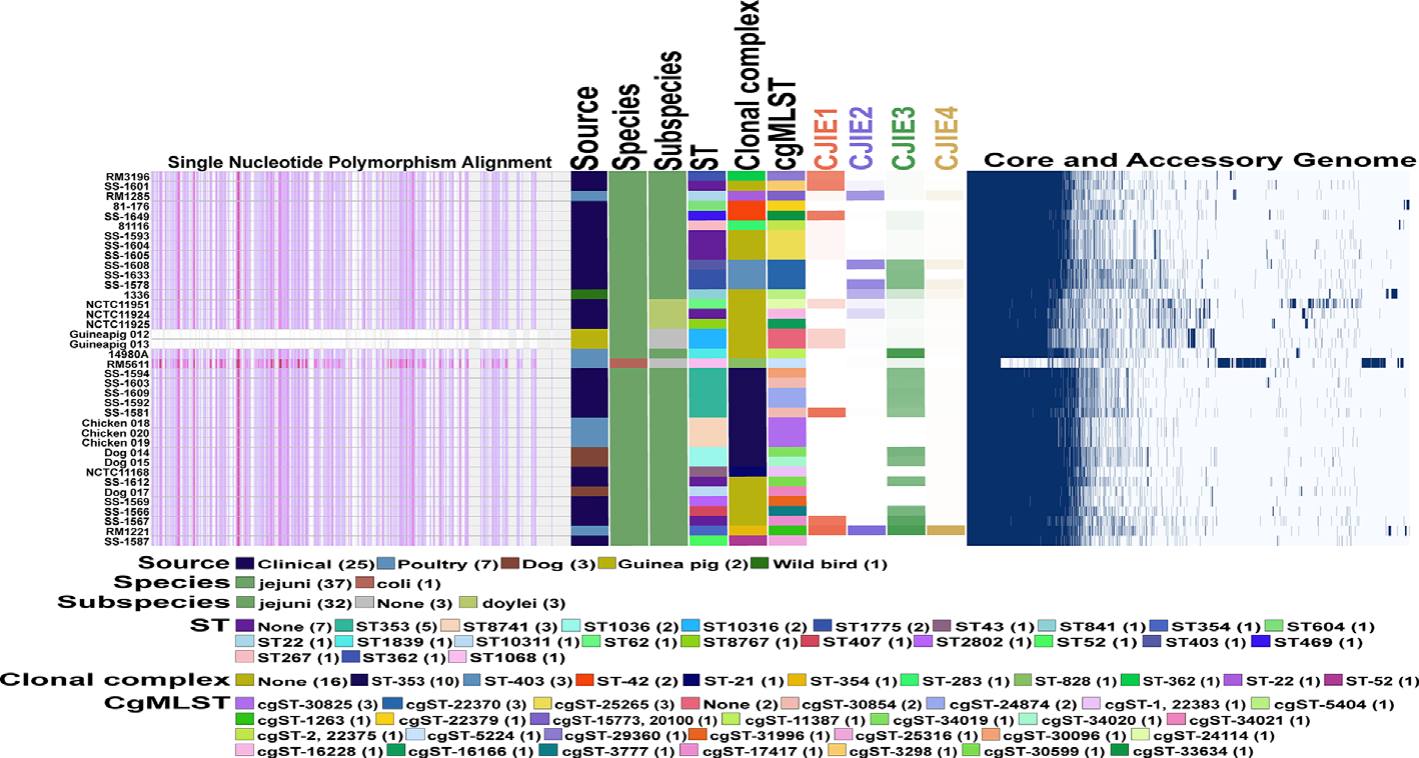
Comparative genomics of Peruvian *Campylobacter jejuni* strains against each other and other *Campylobacter* strains. Core genome phylogeny and single nucleotide polymorphism (SNP) alignments of 38 *Campylobacter* strains using the Harvest software suite. *C. jejuni* str. Guineapig012 was used as the reference strain for analysis. Different characteristics of each of the 38 strains including sequence type (ST) and clonal complex (CC) based on 7 gene multilocus sequence typing (MLST), and the core genome MLST (cgMLST) both using PubMLST database (https://pubmlst.org/) for analysis are shown as follows. Color legend for each section is in the legend in the figure. Intensity mapping of amount of four *Campylobacter jejuni* integrative elements (CJIEs) present in each of the Campylobacter genomes. Percentage of each CJIE present in genome based on nucleotide BLAST (blastn) search against the four full CJIEs present in *Cjj* str. RM1221 using Geneious PRIME software. Visualized comparison of all the core and accessory genes for each of the *Campylobacter* genomes against each other using Roary software.

To characterize genomic differences between the guinea pig isolates and the other *Campylobacter* genomes used in this study, we determined the presence/absence of the four *Campylobacter jejuni* integrated elements (CJIEs) and the genomes on an individual nucleotide level. Conducting core genome single nucleotide polymorphism (SNP) analysis using one of the guinea pig isolates as the reference demonstrates the two isolates from guinea pigs are very closely related. Furthermore, there are significant SNP differences across the entire guinea pig isolate genome compared to the other *Campylobacter* genomes that further supports these strains are distinct to the guinea pig host (**Figure 4**). Additionally, the core and accessory genome mapping further supports the similarity between the two guinea pig isolates and the difference compared to the other 36 *Campylobacter* genomes. Finally, examination of the four CJIEs among the Peruvian strains reveals that CJIE3 is the most common of the elements while CJIE4 is not really found in any of the isolates except *C. jejuni* subsp. *jejuni* str. RM1221. The only one of the four CJIEs that was slightly present in the guinea pig associated isolates were CJIE1, but less than 35% of the integrated element was present in the isolates.

### Core Genes and Genes Unique to Guinea Pig Isolates

An additional pangenome analysis was conducted to further characterize these genomes including confirming the Anvi’o results from the initial pangenome analysis, determining a detailed list of specific core and accessory genes, and identifying those genes unique to the distinct *C. jejuni* strains from guinea pigs. The additional pangenome analysis was conducted using the software programs Roary and Scoary on all the *C. jejuni* genomes (*C. coli* was excluded for this analysis) to overall gain a better appreciation of the significant genomic differences of the strains at the individual gene level. Roary analysis using different percentages (85%-95%) of protein identity cutoff found a range of 1,122 – 918 genes in the core genome (≥95% of the genomes) and accessory genomes ranging from 3,676 – 4,909 genes (**Table 2**). Examination of the gene content differences of the guinea pig distinct isolates with the other *C. jejuni* based on Roary/Scoary analysis demonstrated that there were over 50 genes absent from these isolates, however 55.6% are uncharacterized genes, but does include genes involved in transport, membrane structure, metabolic processes, and translation. Moreover, there were 169 genes that were present only in the guinea pig distinct isolates, but again 84.6% were hypothetical proteins. These Roary/Scoary pangenome results (**Table 3**) were similar to Anvi’o pangenome results (**Figure 3**) that a majority of genes associated only with the guinea pig isolates lacked COG function. However, among genes associated only with the guinea pig isolates there were a few associated with metabolic processes, conjugation, type IV secretion, DNA/nucleotide binding, translation and transmembrane transport (**Table 3**).

**Table 2:** Campylobacter jejuni core genes*.

	95% Identity	90% Identity	85% Identity
**Core genes^1^**	918	1,098	1,122
**Soft core genes^2^**	62	68	61
**Shell genes^3^**	1,134	969	941
**Cloud genes^4^**	3,713	2,875	2,674

^1^Present in 36 ≤ genomes ≤ 37.

^2^Present in 35 ≤ genomes ≤ 36.

^3^Present in 5 ≤ genomes ≤ 35.

^4^Present in < 5 genomes.

*Core genome analysis conducted by Roary software.

**Table 3:** *Campylobacter jejuni* strains associated with guinea pig related genes*.

*Genes present only in C. jejuni strains associated with guinea pig (169)*	
**GO Function/Category**	
Hypothetical protein (143)	Tricarboxylic acid cycle (1)
Propionate catabolic process (1)	Oxidation-reduction process (2)
Conjugation (3)	Nucleotide binding (1)
Oxygen transport (1)	Membrane (2)
Carbohydrate metabolic process (1)	Transcription regulation (1)
Transmembrane transport (1)	DNA binding (1)
Transferase activity (2)	Isomerase activity (2)
DNA repair (1)	Translation (2)
Type IV secretion system (3)	N-acetyltransferase activity (1)
***Genes absent in C. jejuni strains associated with guinea pig (54)***	
**GO Function/Category**	
Hypothetical protein (30)	Translation (2)
Signal transduction (3)	C4-dicarboxylate transport (1)
Oxygen transport (1)	Oxidation-reduction process (4)
Membrane (5)	Siderophore-dependent iron import (1)
Transmembrane transport (2)	DNA repair (1)
Spermidine transport (2)	Transcription regulation (1)
M-molybdopterin cofactor biosynthetic process (1)	

*Based on 90% amino acid identity using Roary/Scoary software for analysis.

### Absence of Selenium Utilization Genes Within *C. jejuni* Isolated From Guinea Pig

**Figure 5:**
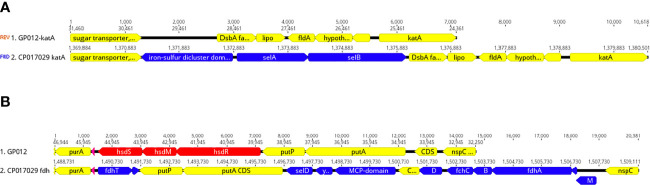
Deletion of selenium utilization and formate dehydrogenase genes. **(A)** Schematic representation demonstrating the deletion of *selAB* from the genomic region adjacent *katA* within the guinea pig isolate and this same region from the genome of *Cjj* strain 14980A that possesses *selAB*. **(B)** Schematic representation demonstrating the deletion of three gene clusters: 1) *fdhTU*, 2) *selDyefD*, and 3) *fdhDCBA* and *fdhM* in a genomic region between *purA* and *nspC* within the guinea pig isolate and this same region in *Cjj* strain 14980A.

Initially, the *selA* gene that encodes the selenocysteine synthase was among the 62 C*. jejuni/C. coli* core genes. However, we were unable to find *selA* in either guinea pig isolate. Further analysis demonstrated that these two genomes were devoid of most of the selenium utilization genes at several locations around the genome including the complete absence of *selAB* (**Figure 5A**)*, selD* and *yefD* (**Figure 5B**), and a partial deletion within *selU.* Each of these gene products are believed to play a role in the translational incorporation of selenocysteine into certain proteins. The *fdhA* gene that encodes a subunit of formate dehydrogenase is one such selenoprotein, but this gene was also completely absent from the *C. jejuni* genomes isolated from guinea pigs (**Figure 5B**). Moreover, these genomes were also missing other genes that are associated with formate dehydrogenase including *fdhB*, *fdhC*, *fdhD, fdhM, fdhT*. and *fdhU* (**Figure 5B**). It should be noted that these additional formate dehydrogenase genes are not selenoproteins.

### Acquisition of the Entner-Doudoroff Pathway

The genomes of the guinea pig distinct isolates both possessed genes for the Entner-Doudoroff pathway, which catalyze the conversion of glucose to pyruvate. Previously, these genes have been identified within certain strains of *Cjj*, *Cjd* and *C. coli* (Vegge et al., 2016). These genes were not among the group of unique genes within the guinea pig *C. jejuni* isolates, as they are also present in the *Cjd* genomes that we examined, but absent from the particular the *Cjj* and *C. coli* in this study.

## Discussion

Although there is a high incidence of campylobacteriosis in LMICs, there is limited understanding about the zoonotic sources of *Campylobacter*. During a collection of samples from possible host animals in the town of Santo Tomas in Amazonian Peru, we were able to isolate *C. jejuni* strains from guinea pig, chicken and dog samples. By analyzing and comparing the WGS of these *C. jejuni* strains with other genomes, we provided evidence that the two strains isolated from guinea pigs from Peru were distinct to that particular host.

The Peruvian Amazon guinea pig isolates had a new unique ST among the more than 95,000 pubMLST samples but shared several MLST alleles with other *C. jejuni* strains previously isolated from guinea pigs in Ecuador (Graham et al., 2016; Vasco et al., 2016). From a neighbor-joining tree based on MLST alleles, most of the guinea pig isolates clustered into two distinct guinea pig specific lineages (**Figure 1**). The cluster that contained the *C. jejuni* strains from guinea pigs isolated in this study and several other guinea pig specific *C. jejuni* strains (Graham et al., 2016; Vasco et al., 2016), is distinct from both *Cjj* and *Cjd* clusters. The guinea pig specific *C. jejuni* lineage is also supported by phylogenetic analysis of 62 core genes and ANI analysis. Again, the results from these two distinct analyses positions the two *C. jejuni* strains from guinea pigs away from other *C. jejuni* strains.

The guinea pig isolates that we sequenced showed evidence of both gene loss and gene addition that help support host adaption. The deletion of multiple genes involved in biosynthesis and utilization of selenocysteine is the most apparent difference when compared to other *C. jejuni* strains. The absence of genes involved in selenium utilization and formate dehydrogenase (a selenocysteine containing enzyme) are not novel occurrences among *Campylobacter*. In fact, the many genomes of the *C. lanienae* clade within the *C. fetus* group are missing many of these same genes (Miller et al., 2017). Guinea pigs are known to have a low selenium dietary requirement with poor selenium dietary reserve. It has been hypothesized that microbial strains that colonize guinea pigs may have selective pressure to favor enzyme pathways that are not selenium dependent (Jensen and Pallauf, 2008). Genes also absent were the *panBCD* genes encoding the vitamin B5 biosynthesis pathway. that are associated with *Cjj* strains that are adapted to cattle (Sheppard et al., 2013). It was suggested that higher levels of vitamin B5 in the diet of poultry, as compared to grass-fed cattle, has made the *panBCD* genes dispensable in poultry specific *Cjj* strains (Sheppard et al., 2013), and this appears to also be true for the isolates from guinea pigs. Finally, the presence of the Entner-Douderoff pathway genes suggest that glucose may be utilized as a carbon source and may also provide fitness advantages to the guinea pig specific *C. jejuni* strains as was observed by Vegge et al. for other *Campylobacters* (Vegge et al., 2016).

Zoonotic host adaption and restriction among *Campylobacter* strains is important to characterize since it aids in the determination of the origin of human infections in settings where there are multiple putative sources of infection. This is important not only in the US and Europe, but across a wider geography and host range in order to inform strategies of disease control in diverse settings. Although this is not a genome-wide association study, the analysis provides initial evidence for genetic factors that lead to the development of a host-specific *C. jejuni*. Microbial adaptation to zoonotic hosts leading to host specialism has been described for the *Campylobacter* isolates from cattle, poultry, and wild birds (Sheppard et al., 2010; Sheppard et al., 2011; Griekspoor et al., 2013; Sheppard et al., 2013; Mourkas et al., 2020). In this study, we identified several host specializing genomic determinants within *C. jejuni* isolates from guinea pigs that may help in source attribution. It appears that the sequestration of *C. jejuni* within the guinea pig host has provided an appropriate barrier and created a particular niche to proliferate this lineage. We only report the genome sequences of two guinea pig host-associated strains, thus, sequencing and phenotypic analysis of additional C. jejuni isolates from guinea pigs will be necessary to verify these strains as host specialists and to determine if they form a new subspecies.

## Data Availability Statement

The datasets presented in this study can be found in online repositories. The names of the repository/repositories and accession number(s) can be found below: https://www.ncbi.nlm.nih.gov/genbank/, JACRSF000000000 JACRSG000000000 JACRSH000000000 JACRSI000000000 JACRSJ000000000 JACRSK000000000 JACRSL000000000 JACRSM000000000.

## Author Contributions

Conceptualization: CP and MK. FS and MK led collection of the isolates. HG sequenced the isolates. Formal Analysis: KC, CP, WM, SH, FS, DT, PB, and P-PY. Original Draft Preparation: CP, KC, FS, and MK. All authors contributed to the article and approved the submitted version.

## Funding

Funding was obtained from the University of Virginia, the Bill and Melinda Gates Foundation (OPP1066146) and from the Sherrilyn and Ken Fischer Center for Environmental Infectious Diseases at the Johns Hopkins School of Medicine to MK. This research was also supported in part by USDA-ARS CRIS project 5325-42000-051-00D. No funding agency had any role in the study design, data collection and analysis, decision to publish, or preparation of the manuscript.

## Conflict of Interest

The authors declare that the research was conducted in the absence of any commercial or financial relationships that could be construed as a potential conflict of interest.

## Abbreviations

LMIC, low- to middle- income country; MLST, multi-locus sequence typing; ST, sequence type; CC, clonal complex; WGS, whole genome sequencing.
